# Hyperandrogenism induces proportional changes in the expression of Kiss-1, Tac2, and DynA in hypothalamic KNDy neurons

**DOI:** 10.1186/s12958-022-00963-w

**Published:** 2022-06-21

**Authors:** Hiroe Okada, Haruhiko Kanasaki, Tuvshintugs Tumurbaatar, Zolzaya Tumurgan, Aki Oride, Satoru Kyo

**Affiliations:** Department of Obstetrics and Gynecology, School of Medicine, Shimane University, 89-1 Enya Cho, Izumo, Shimane 693-8501 Japan

**Keywords:** Testosterone, Androgen, Kisspeptin, Hypothalamus, Prolactin

## Abstract

**Background:**

Kisspeptin released from Kiss-1 neurons in the hypothalamus plays an essential role in the control of the hypothalamic–pituitary–gonadal axis by regulating the release of gonadotropin-releasing hormone (GnRH). In this study, we examined how androgen supplementation affects the characteristics of Kiss-1 neurons.

**Methods:**

We used a Kiss-1-expressing mHypoA-55 cell model that originated from the arcuate nucleus (ARC) of the mouse hypothalamus. These cells are KNDy neurons that co-express neurokinin B (NKB) and dynorphin A (DynA). We stimulated these cells with androgens and examined them. We also examined the ARC region of the hypothalamus in ovary-intact female rats after supplementation with androgens.

**Results:**

Stimulation of mHypoA-55 cells with 100 nM testosterone significantly increased Kiss-1 gene expression by 3.20 ± 0.44-fold; testosterone also increased kisspeptin protein expression. The expression of Tac3, the gene encoding NKB, was also increased by 2.69 ± 0.64-fold following stimulation of mHypoA-55 cells with 100 nM testosterone. DynA gene expression in these cells was unchanged by testosterone stimulation, but it was significantly reduced at the protein level. Dihydrotestosterone (DHT) had a similar effect to testosterone in mHypoA-55 cells; kisspeptin and NKB protein expression was significantly increased by DHT, whereas it significantly reduced DynA expression. In ovary-intact female rats, DTH administration significantly increased the gene expression of Kiss-1 and Tac3, but not DynA, in the arcuate nucleus. Exogenous NKB and DynA stimulation failed to modulate Kiss-1 gene expression in mHypoA-55 cells. Unlike androgen stimulation, prolactin stimulation did not modulate kisspeptin, NKB, or DynA protein expression in these cells.

**Conclusions:**

Our observations imply that hyperandrogenemia affects KNDy neurons and changes their neuronal characteristics by increasing kisspeptin and NKB levels and decreasing DynA levels. These changes might cause dysfunction of the hypothalamic–pituitary–gonadal axis.

## Introduction

Kisspeptin released from Kiss-1 neurons in the hypothalamus plays an essential role in the control of the hypothalamic–pituitary–gonadal (HPG) axis by regulating the release of gonadotropin-releasing hormone (GnRH) from GnRH neurons in the hypothalamus [1]. At present, it is generally agreed that Kiss-1 neurons located in two different areas of the hypothalamus differentially regulate GnRH release. In rodents, Kiss-1 neurons in the anteroventral periventricular nucleus (AVPV) are involved in the surge secretion of GnRH/luteinizing hormone (LH), while those in the arcuate nucleus (ARC) maintain the pulsatile secretion of GnRH [1–3]. Kiss-1 gene expression in the AVPV is upregulated by estradiol (E2), whereas it is repressed by E2 in the ARC [4, 5]. Therefore, Kiss-1-expressing neurons in the AVPV and ARC are believed to be at the center of E2-induced positive and negative feedback control in the HPG axis.

Kiss-1 neurons in the ARC co-express two other neuropeptides, neurokinin B (NKB) and dynorphin A (DynA), and this population is referred to as KNDy neurons, which are preserved across several mammalian species, including rodents, goats, and humans [6]. Loss of function mutations in the gene encoding NKB (Tac3) and its receptor cause hypogonadotropic hypogonadism [7], suggesting that NKB has a stimulatory effect on the HPG axis. On the other hand, DynA is an endogenous opioid peptide that reduces LH pulse frequency in sheep [8]. Thus, KNDy neurons probably generate synchronized oscillatory neuronal activity by receiving excitatory and inhibitory autosynaptic inputs from NKB and DynA, as observed in goats [9].

KNDy neurons in the ARC are influenced by the serum levels of E2, maintain the basal secretion of GnRH, and are involved in E2-induced negative feedback mechanisms. It is plausible that KNDy neurons are affected by androgens because GnRH neurons do not express estrogen receptor α or androgen receptor, but KNDy neurons express both of them [10].

Polycystic ovary syndrome (PCOS) is an endocrine condition that is associated with adverse reproductive, metabolic, and endocrine features in women of reproductive age [11], and hyperandrogenism represents one of the principle traits of PCOS [12]. Indeed, previous animal experiments using monkeys, sheep, and rodents demonstrated that an excess of androgens induces the development of PCOS-like features via androgen receptors [13–15]. Because increased LH pulse frequency driven by an increase in the activity of GnRH neurons is one of the key neuroendocrine aberrations in women with PCOS [16], hypothalamic KNDy neurons, which regulate the activity of GnRH neurons, might be influenced by an excessive androgen milieu. Previous clinical studies demonstrated that serum kisspeptin levels are positively associated with PCOS [17] or androgen levels in obese women with PCOS [18]. However, some reports rejected the usefulness of serum kisspeptin levels as a biomarker for PCOS [19, 20]. The number of kisspeptin- and NKB-immunoreactive neurons in the ARC is increased in a dihydrotestosterone (DHT)-treated rat PCOS model [21], while kisspeptin expression in the hypothalamus is significantly reduced in the same model [22]. Although the importance of androgens and their receptors in the pathogenesis of PCOS neurons has been highlighted, it still unknown how androgens affect KNDy neurons in the hypothalamus.

Kiss-1-expressing mHypoA-55 cells are a model of KNDy neurons that were isolated from the ARC of an adult female mouse. These cells co-express NKB, DynA, and Kiss-1, and Kiss-1 gene expression can be repressed in these cells by E2 under certain experimental conditions [23, 24]. Using these cells, we investigated how androgens directly affect KNDy neurons in the hypothalamus. Furthermore, we examined how androgen supplementation affects the characteristics of KNDy neurons in ovary-intact female rats.

## Materials and Methods

### Materials

The following chemicals and reagents were obtained from the indicated sources: GIBCO fetal bovine serum (Invitrogen, Carlsbad, CA); testosterone, DHT, penicillin–streptomycin, NKB, and prolactin (Sigma-Aldrich Co., St. Louis, MO); and DynA (Cayman Chemical, Ann Arbor, MI).

### Cell culture

mHypoA-55 cells were purchased from Cedarlane (Ontario, Canada). The cells were plated in 35-mm tissue culture dishes and incubated with high-glucose Dulbecco’s modified Eagle’s medium (Sigma-Aldrich Co.) containing 10% heat-inactivated fetal bovine serum and 1% penicillin–streptomycin at 37 °C under a humidified atmosphere of 5% CO_2_ in air. After 24 h, the medium was changed to high-glucose Dulbecco’s modified Eagle’s medium containing 1% heat-inactivated fetal bovine serum and 1% penicillin–streptomycin, and the cells were incubated without (control) or with various concentrations of test reagents (testosterone, DHT, NKB, DynA, and prolactin) for 24 h.

### RNA preparation, reverse transcription, and quantitative real-time polymerase chain reaction

Total RNA was extracted from the cells using TRIzol-LS (Invitrogen). To obtain cDNA, 1.0 µg total RNA was reverse-transcribed using an oligo-dT primer (Promega, Madison, WI) and prepared using a First-Strand cDNA Synthesis Kit (Invitrogen) in reverse transcription buffer. The preparation was supplemented with 10 mM dithiothreitol, 1 mM of each dNTP, and 200 U RNase inhibitor/human placenta ribonuclease inhibitor (Cat. No. 2310; Takara, Tokyo, Japan) in a final volume of 10 µL. The reaction was incubated at 37 °C for 60 min. Kiss-1, NKB, and DynA mRNA levels were determined by using quantitative real-time (RT)-PCR (ABI Prism 7000; Perkin-Elmer Applied Biosystems, Foster City, CA) according to the manufacturer’s protocol (User Bulletin No. 2) as well as Universal ProbeLibrary probes and Fast Start Master Mix (Roche Diagnostics, Mannheim, Germany). Using specific primers for Kiss-1 (forward: 5′-AGCTGCTGCTTCTCCTCTGT-3′ and reverse: 5′-GCATACCGCGATTCCTTTT-3′), NKB (forward: 5′-GACTGGCCCTCTCTGAGTTC-3′ and reverse: 5′-ACGGAGGCAGCTGATAGAGA-3′), and DynA (forward: 5′-GGATTTGGTAGCCCTCGTCG-3′ and reverse: 5′-AATCCTCACTGCACACAGGG-3′), the simultaneous measurement of Kiss-1, NKB, and DynA mRNA was performed. GAPDH mRNA was used to normalize the amount of cDNA added per sample. For each set of primers, a no-template control was included. Thermal cycling conditions were as follows: 10 min denaturation at 95 °C, followed by 40 cycles of 95 °C for 15 s and 60 °C for 1 min. Reactions were followed by melting curve analysis (55 °C–95 °C).

To determine PCR efficiency, a tenfold serial dilution of cDNA was performed as described previously [25]. The PCR conditions were optimized to generate > 95% efficiency, and only those reactions with between 95 and 105% efficiency were included in subsequent analyses. Relative differences in cDNA concentrations between the baseline and experimental conditions were calculated using the comparative threshold cycle (Ct) method [26]. Briefly, for each sample, ΔCt was calculated to normalize expression to the internal control (GAPDH) by using the following equation: ΔCt = ΔCt(gene) – Ct(GAPDH). To determine differences between the experimental and control conditions, ΔΔCt was calculated as ΔCt(sample) − ΔCt(control). Relative mRNA levels were calculated using the following equation: fold difference = 2^ΔΔCt^.

### Western blot analysis

Cell extracts were lysed on ice with RIPA buffer (phosphate-buffered saline, 1% NP-40, 0.5% sodium deoxycholate, and 0.1% sodium dodecyl sulfate [SDS]) containing 0.1 mg/mL phenylmethylsulfonyl fluoride, 30 mg/mL aprotinin, and 1 mM sodium orthovanadate, scraped for 20 s, and centrifuged at 14,000 × *g* for 10 min at 4 °C. The protein concentrations of cell lysates were measured using the Bradford method. Denatured protein (20 µg) was resolved by 10% SDS–polyacrylamide gel electrophoresis (PAGE) according to standard protocols and transferred to polyvinylidene difluoride membranes (Hybond-P PVDF; Amersham Biosciences, Little Chalfont, UK), which were blocked for 2 h at room temperature in Blotto (5% milk in Tris-buffered saline). The membranes were incubated with an anti-kisspeptin antibody (1:250 dilution; Abcam, Cambridge, UK), anti-NKB antibody (1:250; Abcam), or anti-DynA antibody (1:1,000; Abcam) in Blotto overnight at 4 °C and washed three times at 10 min per wash with Tris-buffered saline/1% Tween. A subsequent incubation with horseradish peroxidase (HRP)-conjugated antibodies was performed for 1 h at room temperature in Blotto, and additional washes were performed as needed. Following enhanced chemiluminescence detection (Amersham Biosciences), the membranes were exposed to X-ray film (Fujifilm, Tokyo, Japan). After strip washing (Restore Buffer; Pierce Chemical Co., Rockford, IL), the membranes were reprobed with an anti-β-actin antibody (1:15,000 dilution; Abcam) for 1 h at room temperature and incubated with HRP-conjugated secondary antibodies before the procedure was continued as described above. When the expression levels were compared after stimulation, the films were analyzed by densitometry, and the intensity of the target protein was normalized to that of β-actin to correct for protein loading.

### Animal experiments

Six-week-old female Wistar rats (The Jackson Laboratory Japan Inc., Yokohama, Japan) were maintained under a 12-h light/dark cycle at 20 °C–25 °C with food (CE-2; CLEA Japan, Tokyo, Japan) and water available ad libitum. Rats were housed 2 per cage. Vaginal smears were taken daily, and only rats showing at least three consecutive regular estrus cycles were included in the study. The rats received a daily subcutaneous injection of DHT (5 mg/kg/day) to produce a supraphysiological androgen level in vivo, based on a previous study [27], or a placebo control in 160 μL sesame oil (Fujifilm, Tokyo, Japan) for 7 days. Then, the rats were euthanized while under isoflurane anesthesia and the whole brain was removed. Hypothalamic tissues containing the ARC were extracted and used for quantitative RT-PCR analysis. This protocol was approved by the ethics committee of the Experimental Animal Center for Integrated Research at Shimane University (IZ31-51).

### Statistical analysis

All experiments were repeated independently at least three times. Each experiment in each experimental group was performed using duplicate samples. When mRNA expression was determined, two samples were assayed in duplicate. From four sets of data, we calculated the mean ± standard error of the mean (SEM). Averages from three independent experiments were statistically analyzed. Data are expressed as mean ± SEM values. Statistical analysis was performed using one-way analysis of variance with Bonferroni’s post hoc test or Student’s *t*-test. *P* < 0.05 was considered statistically significant.

## Results

### Effect of testosterone on kisspeptin expression in mHypoA-55 cells

**Fig. 1:**
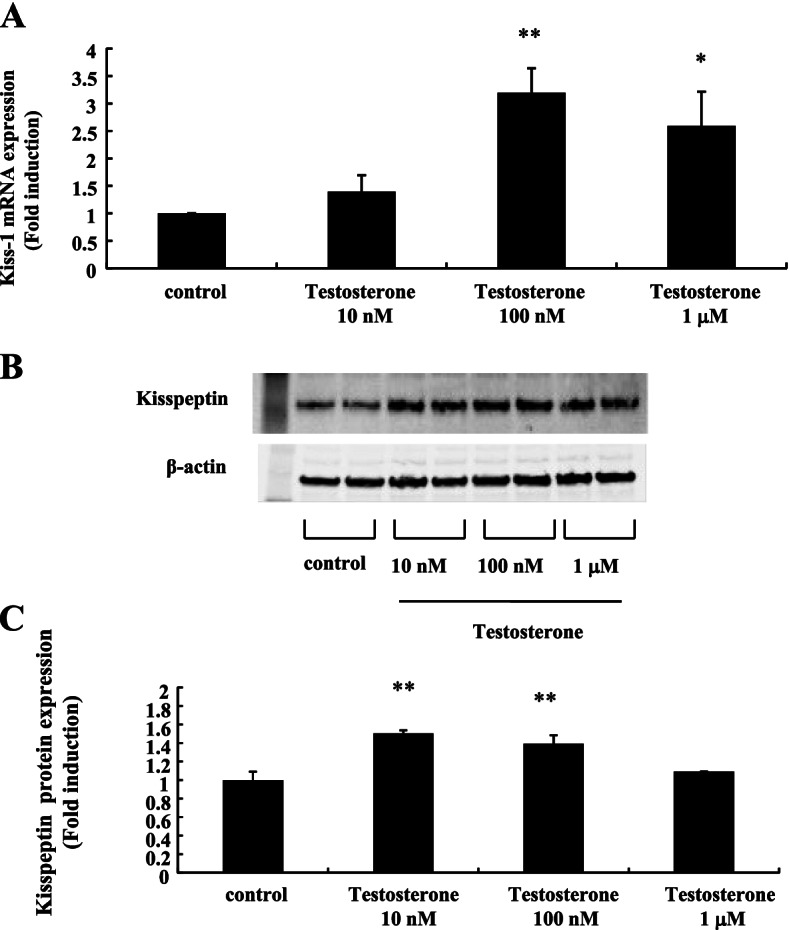
Effect of testosterone on Kiss-1 gene and kisspeptin protein expression in mHypoA-55 cells. mHypoA-55 cells were stimulated with the indicated concentrations of testosterone for 24 h. **A** After stimulation, mRNA was extracted and reverse transcribed, and Kiss-1 mRNA levels were measured by quantitative RT-PCR. Samples for each experimental group were run in duplicate and normalized to the mRNA levels of GAPDH as a housekeeping gene. The results are expressed as fold induction over unstimulated cells and presented as the mean ± SEM of three independent experiments. **P < 0.01, *P < 0.05 vs. control. **B** After stimulation, lysates (20  g protein) from mHypoA-55 cells were analyzed by SDS-PAGE followed by immunoblotting and incubation with anti-kisspeptin antibodies. The bands were visualized using an HRP-conjugated secondary antibody. **C** Scanning densitometry of bands was performed using ImageJ to determine differences in kisspeptin protein expression, with normalization to β-actin levels. Results are expressed as fold stimulation over the unstimulated group/control. Values are the mean ± SEM of fold stimulation from independent experiments. **P < 0.01 vs. control

To examine the direct effect of testosterone on mHypoA-55 KNDy neurons, mHypoA-55 cells were stimulated with increasing concentrations of testosterone and Kiss-1 gene and kisspeptin protein levels were determined. When mHypoA-55 cells were cultured with 100 nM testosterone for 24 h, Kiss-1 gene expression was significantly increased by 3.20 ± 0.44-fold compared to non-stimulated control cells (Fig. 1A). Kisspeptin protein expression was also significantly increased by 10 nM testosterone stimulation to 1.5 ± 0.03-fold (Fig. 1B and C).

### Effect of testosterone on NKB expression in mHypoA-55 cells

**Fig. 2:**
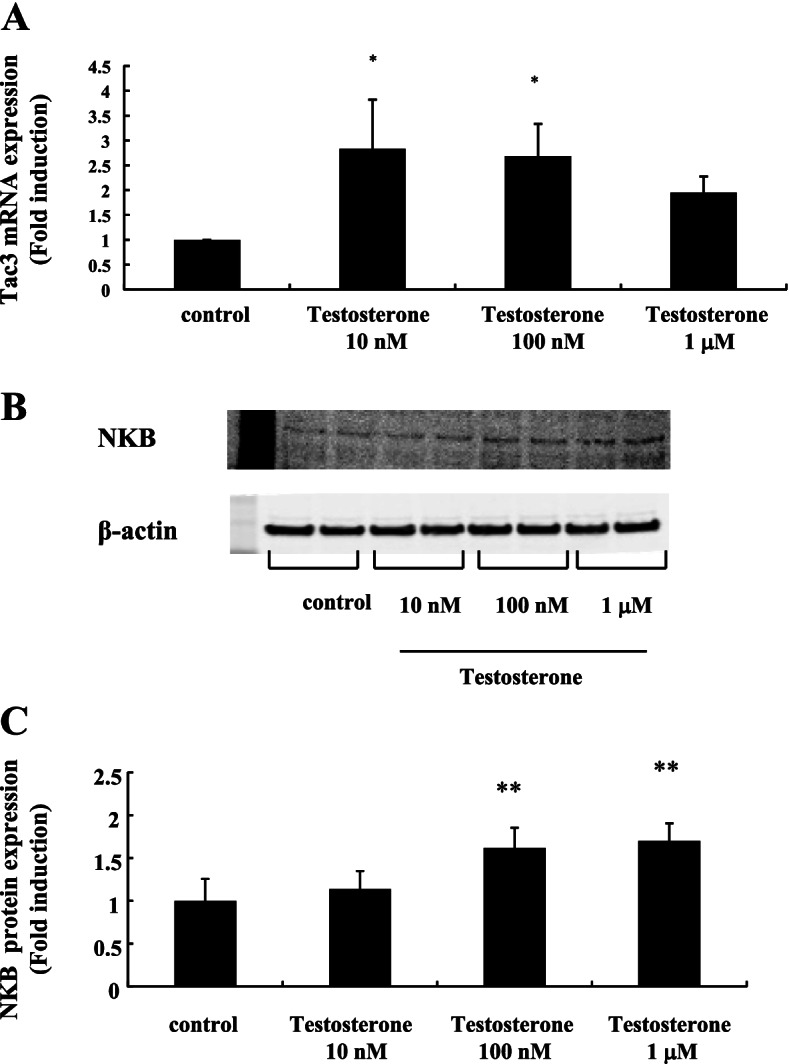
Effect of testosterone on Tac3 gene and NKB protein expression in mHypoA-55 cells. mHypoA-55 cells were stimulated with the indicated concentrations of testosterone for 24 h. **A** After stimulation, mRNA was extracted and reverse transcribed. Tac3 mRNA levels were measured by quantitative RT-PCR. Samples for each experimental group were run in duplicate and normalized to the mRNA levels of GAPDH as a housekeeping gene. The results are expressed as fold induction over unstimulated cells and presented as the mean ± SEM of three independent experiments. *P < 0.05 vs. control. **B** After stimulation, lysates (20  g protein) from mHypoA-55 cells were analyzed by SDS-PAGE followed by immunoblotting and incubation with anti-NKB antibodies. The bands were visualized using an HRP-conjugated secondary antibody. **C** Scanning densitometry of bands was performed using ImageJ to determine differences in kisspeptin protein expression, with normalization to β-actin levels. Results are expressed as fold stimulation over the unstimulated group/control. Values are the mean ± SEM of fold stimulation from independent experiments. **P < 0.01 vs. control

Next, we examined how testosterone affected NKB expression in mHypoA-55 cells. Tac3 expression, the gene encoding NKB, was significantly increased by 2.84 ± 0.98-fold and 2.69 ± 0.64-fold by 10 nM and 100 nM testosterone, respectively (Fig. 2A). NKB protein expression was also increased by 100 nM testosterone to 1.62 ± 0.64-fold (Fig. 2B and C).

### Effect of testosterone on DynA expression in mHypoA-55 cells

**Fig. 3:**
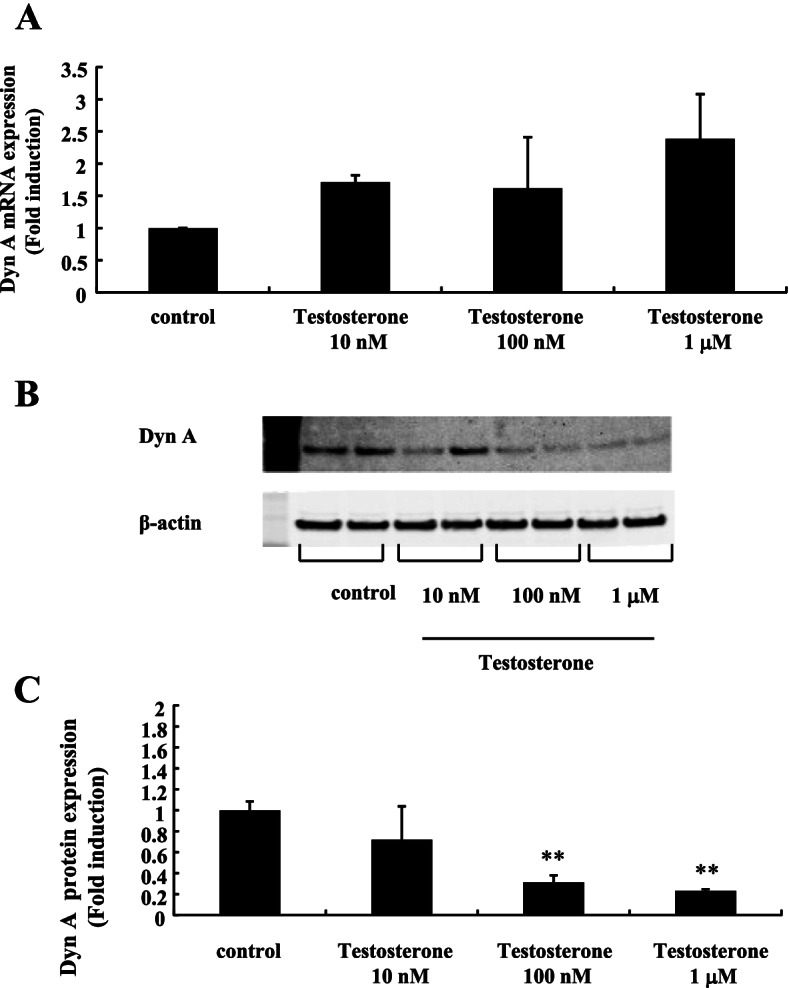
Effect of testosterone on DynA expression in mHypoA-55 cells. mHypoA-55 cells were stimulated with the indicated concentrations of testosterone for 24 h. **A** After stimulation, mRNA was extracted and reverse transcribed. DynA mRNA levels were measured by quantitative RT-PCR. Samples for each experimental group were run in duplicate and normalized to the mRNA levels of GAPDH as a housekeeping gene. The results are expressed as fold induction over unstimulated cells and presented as the mean ± SEM of three independent experiments. **B** After stimulation, lysates (20  g protein) from mHypoA-55 cells were analyzed by SDS-PAGE followed by immunoblotting and incubation with anti-DynA antibodies. The bands were visualized using an HRP-conjugated secondary antibody. **C** Scanning densitometry of bands was performed using ImageJ to determine differences in DynA protein expression, with normalization to β-actin levels. Results are expressed as fold stimulation over the unstimulated group/control. Values are the mean ± SEM of fold stimulation from independent experiments. **P < 0.01 vs. control

DynA expression in mHypoA-55 cells seemed to be decreased by testosterone stimulation. Although we could not detect significant changes in DynA mRNA expression (Fig. 3A), western blot analysis using an anti-DynA antibody revealed that its expression was significantly decreased by 100 nM and 1  M testosterone stimulation by 70%–80% compared to non-stimulated cells (Fig. 3B and C).

### Effect of DHT on kisspeptin, NKB, and DynA expression in mHypoA-55 cells

**Fig. 4:**
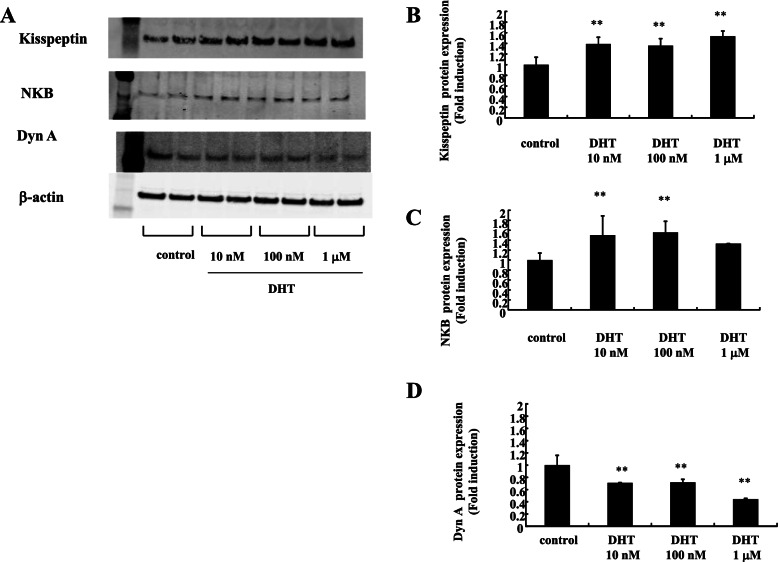
Effect of DHT on the expression of kisspeptin, NKB, and DynA in mHypoA-55 cells. (A) mHypoA-55 cells were stimulated with the indicated concentrations of DHT for 24 h, and lysates (20  g protein) from the treated cells were analyzed by SDS-PAGE followed by immunoblotting and incubation with antibodies against kisspeptin, NKB, and DynA. The bands were visualized using an HRP-conjugated secondary antibody. Scanning densitometry of bands was performed using ImageJ to determine differences in kisspeptin (A), NKB (B), and DynA (C) expression, with normalization to β-actin levels. Results are expressed as fold stimulation over the unstimulated group/control. Values are the mean ± SEM of fold stimulation from independent experiments. **P < 0.01 vs. control

DHT is a potent endogenous androgen synthesized from testosterone by 5α-reductase. To examine the effect of DHT on mHypoA-55 KNDy neurons, the cells were stimulated with DHT for 24 h and kisspeptin, NKB, and DynA expression was determined by western blotting analysis (Fig. 4). Kisspeptin (Fig. 4A and B) and NKB (Fig. 4A and C) expression was significantly increased by DHT stimulation. Conversely, DynA expression was significantly reduced by DHT stimulation (Fig. 4A and D). These effects were the same as those observed for testosterone stimulation.

### Effect of DHT administration on ovary-intact female rats

**Fig. 5:**
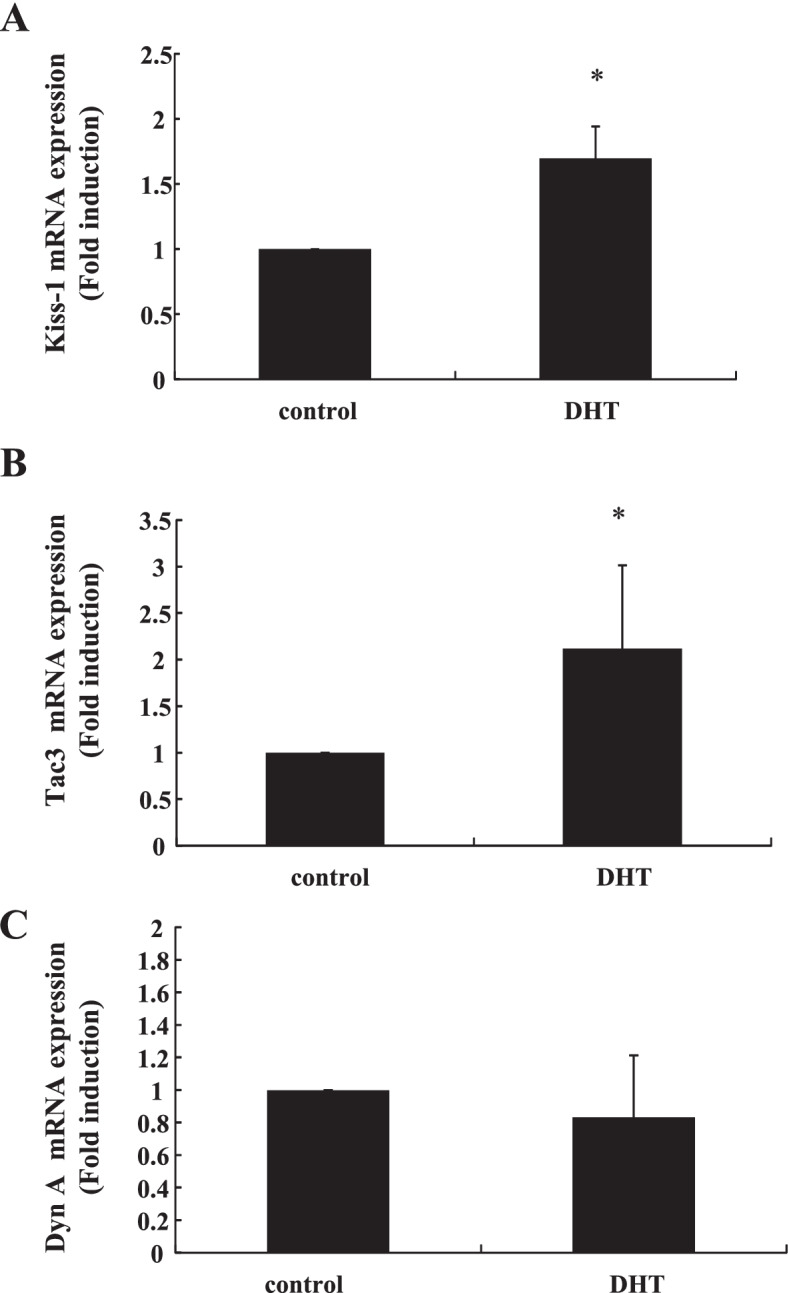
Effect of DHT supplementation on Kiss-1, Tac3, and DynA gene expression in ovary-intact female rats. Six-week-old female rats were injected subcutaneously with DHT (5 mg/kg/day) daily for 7 days. After the rats were euthanized, hypothalamic tissues containing the ARC were removed from control (*n* = 4) and DHT-treated rats (*n* = 4), and mRNA was extracted from these tissues and reverse transcribed. Kiss-1, Tac3, and DynA mRNA levels were measured by quantitative RT-PCR. Samples for each experimental group were run in duplicate and normalized to the mRNA levels of GAPDH as a housekeeping gene. The results are expressed as fold induction over control and presented as the mean ± SEM. *P < 0.05 vs. control

To examine the effect of supraphysiological androgens in ovary-intact female rats, DHT (5 mg/kg/day) was administered to rats once a day for 7 days. Then, brain tissues from the ARC were extracted and the expression of Kiss-1, Tac3, and DynA genes was compared with non-treated rats. Kiss-1 gene expression in the ARC was significantly increased by DHT administration (1.70 ± 0.25-fold) (Fig. 5A). Similarly, Tac3 gene expression in this area was also upregulated by DHT treatment compared to non-treated rats (2.12 ± 0.89-fold) (Fig. 5B). In contrast, DynA gene expression in this area was unchanged following DHT administration (Fig. 5C).

### Effect of NKB and DynA stimulation on Kiss-1 gene expression in mHypoA-55 cells

**Fig. 6:**
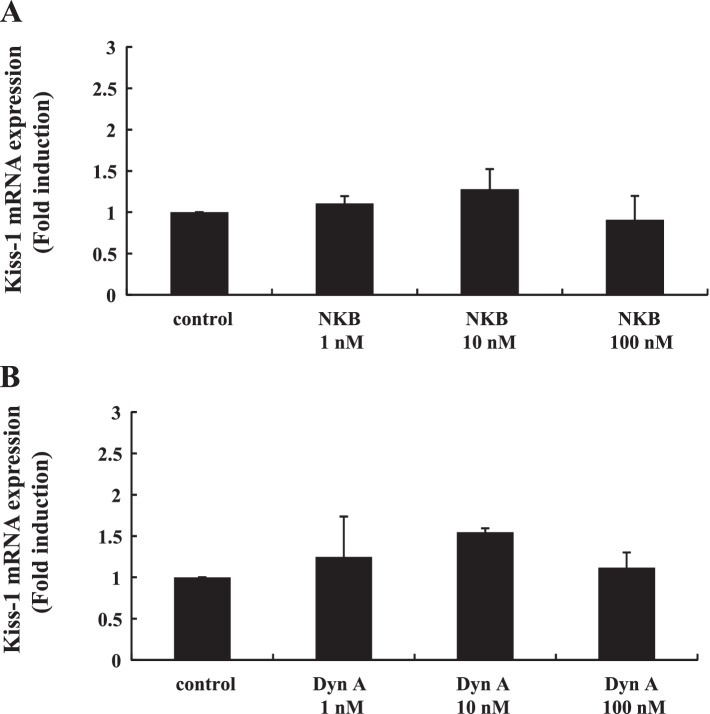
Effect of NKB and DynA stimulation on Kiss-1 gene expression in mHypoA-55 cells. mHypoA-55 cells were stimulated with the indicated concentrations of NKB or DynA for 24 h. (A) After stimulation, mRNA was extracted and reverse transcribed, and Kiss-1 mRNA levels were measured by quantitative RT-PCR. Samples for each experimental group were run in duplicate and normalized to the mRNA levels of GAPDH as a housekeeping gene. The results are expressed as fold induction over unstimulated cells and presented as the mean ± SEM of three independent experiments

To examine the direct effect of NKB and DynA stimulation on Kiss-1-expressing neurons, mHypoA-55 cells were stimulated with increasing concentrations of NKB and DynA for 24 h, and then Kiss-1 gene expression was determined. Neither NKB nor DynA significantly altered Kiss-1 gene expression in mHypoA-55 ARC cells (Fig. 6A and B).

### Effect of prolactin on Kiss-1, NKB, and DynA protein expression in mHypoA-55 ARC cells

**Fig. 7:**
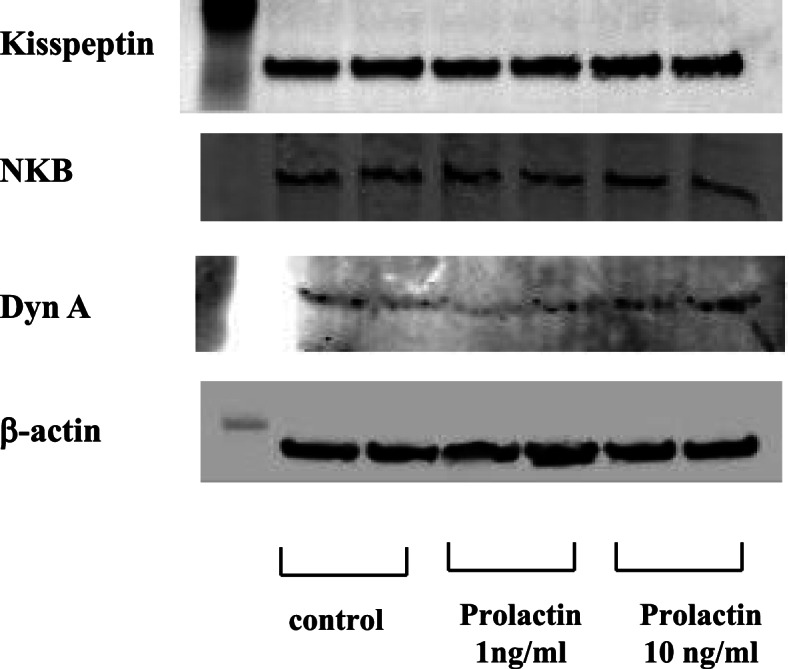
Effect of prolactin on kisspeptin, NKB, and DynA expression in mHypoA-55 cells. (A) mHypoA-55 cells were stimulated with the indicated concentrations of prolactin for 24 h. Lysates (20  g protein) from mHypoA-55 cells were analyzed by SDS-PAGE followed by immunoblotting and incubation with antibodies against kisspeptin, NKB, and DynA. The bands were visualized using an HRP-conjugated secondary antibody

As observed for androgens, prolactin serum levels tend to be elevated in patients with PCOS. Finally, we examined how the expression of kisspeptin, NKB, and DynA in KNDy neurons was changed by prolactin stimulation. mHypoA-55 ARC KNDy cells were stimulated with 1 or 10 ng/mL prolactin for 24 h. Western blotting analysis showed that neither concentration of prolactin modulated the protein expression levels of kisspeptin, NKB, and DynA (Fig. 7).

## Discussion

In this study, we examined the effects of androgens on the characteristics of KNDy neurons in the ARC region of the hypothalamus. As a KNDy neuron cell model, we used mHypoA-55 cells, which originated from the murine ARC. It is well documented that kisspeptin stimulates LH release via GnRH neurons, while DynA inhibits the pulse frequency of GnRH [6]. Although the effect of NKB on gonadotropin release is controversial, Wakabayashi et al. (2010) performed experiments using goats and demonstrated that NKB induced multiple-unit activity of the neurons in the ARC and increased LH pulse frequency. They also demonstrated that the DynA-inhibited multiple-unit activity of the neurons in this area was associated with a concomitant decrease in pulsatile LH secretion.

Hyperandrogenism is a medical condition characterized by high serum levels of androgens in females. Because hyperandrogenism is frequently observed in women with PCOS, who are characterized by anovulation associated with abnormal gonadotropin secretion, which is reflected by abnormal GnRH pulse frequency, we speculated that an excess of androgens would have an effect on KNDy neurons, which govern the secretory patterns of GnRH. mHypoA-55 cells originated from mouse hypothalamic Kiss-1 neurons located in the ARC. These cells were defined as KNDy neurons because they express NKB (Tac3), DynA, and Kiss-1, and show a similar response to E2 stimulation on Kiss-1 gene expression [23, 24]. Testosterone stimulation induced Kiss-1 gene and kisspeptin protein expression with a concomitant increase in Tac3 gene expression and NKB protein levels. The more potent androgen DHT had a similar effect on Kiss-1 and Tac3 gene expression in these cells. These observations are consistent with previous animal experiments showing that the administration of DHT to rats increases kisspeptin and NKB expression in the ARC [21]. On the other hand, DynA protein expression seemed to be repressed in the presence of testosterone and DHT in our cell model. Thus, it is plausible that androgens could increase kisspeptin and NKB levels, while decreasing DynA expression in KNDy neurons.

To confirm the effect of androgens on KNDy neurons in vivo, DHT was administered to rats and we examined whether kisspeptin, NKB, and DynA expression levels were changed in the ARC. In this animal experiment, we wanted to examine the changes in KNDy neurons under the condition of hyperandrogenism, which is frequently observed in patients with PCOS. Although the control of follicular development is disrupted in patients with PCOS, their ovarian reserve, which is reflected by high anti-Müllerian hormone levels, is sufficient. Therefore, we administered DHT to ovary-intact female rats and examined the changes in KNDy neurons. Kiss-1 and Tac3 gene expression was significantly increased in the ARC of ovary-intact female rats; however, DynA gene expression was unchanged. These observations were quite similar to the gene expression pattern observed in the mHypoA-55 ARC cell model. DynA protein expression in mHypoA-55 cells was clearly decreased by testosterone or DHT treatment, although DynA gene expression was not inhibited significantly by testosterone stimulation. In our in vivo rat study, we could not determine the protein expression levels of kisspeptin, NKB, and DynA because the amount of tissue obtained from the ARC was very limited and could not be used for western blotting analysis. Thus, although DHT treatment did not change DynA gene expression in ovary-intact rats, it is reasonable to assume that its protein expression might be decreased, as observed in mHypoA-55 cells.

Numerous findings support a causative role for an excess of androgens in driving the pathogenesis of PCOS [28]. Genetic modification of androgen receptors in mice, which exhibit haplo- or complete androgen receptor insufficiency, protects against the development of PCOS features [29, 30]. Osuka et al. (2017) reported that kisspeptin- and NKB-immunoreactive neurons are increased in a prenatal DHT-exposed rat model of PCOS. In their prenatal DHT-treated model, pregnant dams were injected subcutaneously with DHT once and then a subcutaneous DHT pellet was implanted into the pups for approximately 3 months. They did not examine the alteration of kisspeptin and NKB expression in postnatal DHT-treated rats because basal LH levels were not increased. In our in vivo experiments, the rats were injected subcutaneously with DHT daily for only 7 days. Ovarian morphology was not altered under this short DHT treatment regimen, and we did not intend to develop a rat PCOS model. Nevertheless, our current study using the mHypoA-55 ARC cell model and ovary-intact female rats indicated that androgens have the ability to increase kisspeptin and NKB levels, and probably decrease DynA expression in KNDy neurons in the ARC. We showed the possibility that hyperandrogenemia in PCOS could secondarily induce changes in KNDy neurons, but it is still unknown whether these changes underlie the pathogenesis of PCOS. A recent study by Sucquart et al. (2021) demonstrated that targeted antagonism of NKB signaling ameliorates the metabolic features of PCOS. These observations suggest that the androgen-induced increase in NKB expression could represent the pathogenic mechanism of PCOS. In addition, Esparza et al. (2020) reported that the expression of kisspeptin and NKB within the ARC was increased in a mouse model of PCOS that was developed using chronic letrozole treatment. Letrozole-treated PCOS models reportedly exhibit disrupted estrous cyclicity, increased ovarian weight and cysts, hyperandrogenemia, and anovulation [31, 32]. Considering previous observations, morphological changes in the ovary and subsequent changes in metabolic features might alter the expression levels of kisspeptin, NKB, and DynA in the hypothalamus.

Kisspeptin and NKB both stimulate GnRH pulse output, while DynA inhibits KNDy neuron firing during pulsation [9, 33, 34]. Thus, we speculated that changes in the proportion of NKB and DynA would have an effect on kisspeptin production and release from KNDy neurons. Although NKB and DynA are considered to act in an autocrine/paracrine manner on KNDy neurons, exogenous stimulation by NKB and DynA did not alter Kiss-1 gene expression in mHypoA-55 cells. Considering this observation, the androgen-induced proportional changes in NKB, DynA, and kisspeptin expression within KNDy neurons may alter the secretory pattern of kisspeptin and eventually alter the pattern of pulsatile GnRH release, resulting in hyperactive LH secretion. In addition to hyperandrogenemia, hyperprolactinemia is most common etiology of anovulation in women [35]. Although previous reports demonstrated the suppressive effect of prolactin on KNDy neurons in rats [36, 37], we did not observe any alteration in the proportional expression of kisspeptin, NKB, and DynA in mHypoA-55 KNDy neurons.

Gonadal steroid hormones play an important role in determining sex differences in the brain as well as in behavior [38]. As previously described, prenatal administration of androgens induces the PCOS phenotype, but it also induces masculinization and reduces sexual behavior in females [39]. Kisspeptin neurons also have roles in sexual and emotional processing [40] as well as metabolism [41]. Proportional changes in KNDy neurons induced by androgens might influence a broad range of physiologic activities.

## Conclusions

In this study, we observed that testosterone and DHT had a direct effect on the mHypoA-55 KNDy neuron cell model. Similar results were obtained with in vivo rat experiments. Androgens induced proportional changes in the expression of kisspeptin, NKB, and DynA in KNDy neurons. This increased expression of kisspeptin and NKB and decreased expression of DynA might alter the characteristics of these neurons and ultimately lead to dysfunction of the HPG axis.

## Data Availability

The datasets used and/or analyzed during this study are available from corresponding authors on reasonable request.

## Abbreviations

HPG axis: Hypothalamic-pituitary gonadal axis
GnRH: Gonadotropin-releasing hormone
LH: Luteinizing hormone
AVPV: Anteroventral periventricular nucleus
ARC: Arcuate nucleus
KNDy neurons: Kisspeptin-neurokinin B-dynorphin A neurons
PCOS: Polycystic ovary syndrome
